# Highly Diversified *Pandoraea pulmonicola* Population during Chronic Colonization in Cystic Fibrosis

**DOI:** 10.3389/fmicb.2017.01892

**Published:** 2017-10-06

**Authors:** Chloé Dupont, Fabien Aujoulat, Raphaël Chiron, Pauline Condom, Estelle Jumas-Bilak, Hélène Marchandin

**Affiliations:** ^1^Equipe Pathogènes Hydriques, Santé, Environnements, UMR 5569 Hydrosciences Montpellier, U.F.R des Sciences Pharmaceutiques et Biologiques and Université Montpellier, Montpellier, France; ^2^Centre de Ressources et de Compétences pour la Mucoviscidose, Hôpital Arnaud de Villeneuve, Centre Hospitalier Universitaire de Montpellier, Montpellier, France; ^3^Laboratoire d’Hygiène Hospitalière, Hôpital Saint-Eloi, Centre Hospitalier Universitaire de Montpellier, Montpellier, France; ^4^Service de Microbiologie, Hôpital Carémeau, Centre Hospitalier Universitaire de Nîmes, Nîmes, France; ^5^Laboratoire de Bactériologie, Hôpital Arnaud de Villeneuve, Centre Hospitalier Universitaire de Montpellier, Montpellier, France

**Keywords:** cystic fibrosis, *Pandoraea*, diversity, persistence, biofilm, motility, antimicrobial resistance, virulence

## Abstract

Several environmental bacteria are considered as opportunistic pathogens in cystic fibrosis (CF) and are able to persistently colonize the CF respiratory tract (CFRT). Beside *Pseudomonas aeruginosa* and *Burkholderia cepacia* complex, *Pandoraea* spp. are defined as pathogenic. During chronic colonization, adaptive evolution and diversified population have been demonstrated, notably for *P. aeruginosa*. However, the persistence of *Pandoraea* in the CFRT remains largely unexplored. We studied genomic and phenotypic traits of *Pandoraea pulmonicola* isolates successively recovered from the airways of a single CF patient and relate the results to qualitative and quantitative evolution of other cultivable pathogens and to patient clinical status. A total of 31 isolates recovered from 18 sputum samples over a 7-year period in a single CF patient were studied. Genome dynamics was assessed by pulsed-field gel electrophoresis, ERIC-PCR fingerprinting and 16S rRNA gene PCR-temporal temperature gel electrophoresis. Phenotypic features included antimicrobial susceptibility, motility, biofilm production, and virulence in *Caenorhabditis elegans* model. Variability was observed for all the characteristics studied leading to highly diversified patterns (24 patterns) for the 31 clonally related isolates. Some of these modifications, mainly genomic events were concomitantly observed with CFRT microbiota composition shifts and with severe exacerbations. The diversity of *P. pulmonicola* population studied, observed for isolates recovered from successive samples but also within a sample suggested that existence of a diversified population may represent a patho-adaptive strategy for host persistence in the heterogeneous and fluctuating CFRT environment.

## Introduction

The genus *Pandoraea* was described in 2000, it belongs to the family *Burkholderiaceae* and comprises 10 species mainly cultured from sputum of cystic fibrosis (CF) patients or soil (Coenye et al., 2000). Currently, 20 *Pandoraea* genomes are totally sequenced, including a unique *Pandoraea pulmonicola* genome. *Pandoraea* species are considered as emerging pathogens and infection led to the production of high levels of antibodies, and to a worsened CF lung disease (Jørgensen et al., 2003; Mahenthiralingam, 2014; Degand et al., 2015; Martina et al., 2017). After first colonization, *Pandoraea* spp. were able to chronically colonize the CF respiratory tract (CFRT) (Fernández-Olmos et al., 2012; Kokcha et al., 2013; Pugès et al., 2015; Martina et al., 2017), were transmissible between patients (Jørgensen et al., 2003; Degand et al., 2015) and can produce severe lung diseases and bacteremia (Pimentel and MacLeod, 2008; Kokcha et al., 2013). The pathogenicity appears mainly supported by a pro-inflammatory response induction significantly greater than with *Pseudomonas aeruginosa* (Caraher et al., 2008; Costello et al., 2014) and the treatment may be complicated by multidrug resistance conferred by carbapenem-hydrolyzing oxacillinases (Caraher et al., 2008). *P. pulmonicola* showed an ability to invade human lung epithelial cells, not shared by other *Pandoraea* species (Caraher et al., 2008) and was the most virulent species, being as virulent as *Burkholderia cenocepacia* in the *Galleria mellonella* larvae model (Costello et al., 2011). The potential involvement of *Pandoraea* in complex interactions between microorganisms within the CF airways was also suggested (Costello et al., 2014).

During long-term colonization, adaptive evolution was found in most CF pathogens and was particularly studied for *P. aeruginosa* and *Burkholderia* spp. (Silva et al., 2011). In *P. aeruginosa*, evolutionary adaptation and phenotype diversification occur during CFRT colonization (Hauser et al., 2011; Winstanley et al., 2016 for reviews). These variations generally lead to decreased immunogenicity and virulence while antibiotic resistance globally increases. In addition, some studies revealed a diversified population within a sample and suggested that this diversity may also represent an adaptive strategy for host persistence (Lieberman et al., 2014; Winstanley et al., 2016).

Adaptive behavior of other CF pathogens has received less attention although an increased virulence has been suggested during chronic colonization for *P. pulmonicola* (Costello et al., 2011). Here, we studied the diversity of genomic and phenotypic traits of *P. pulmonicola* isolates recovered from the airways of a CF patient over a 7-year period. Results were related to the overall dynamics of the cultivable CFRT pathogens and to the patient clinical status.

## Materials and Methods

### Patient, Bacterial Isolates, Clinical and Follow-Up Microbiological Data

*Pandoraea* isolates were recovered from 18 sputum samples from a single CF patient attending the CF center of the University Hospital of Montpellier, South of France. All samples were analyzed according to national recommendations (Société Française de Microbiologie, 2015) and the initial isolate was identified by 16S rRNA gene sequencing. Isolates were named with numbers (1–18) according to chronological sampling order, and isolates with different colonial morphotypes in a sample were named with the same number followed by apostrophe(s). Selected co-colonizing *P. aeruginosa* (Pa13 isolated in September 2013) and methicillin-resistant *Staphylococcus aureus* (Sa14 isolated in January 2014) isolated at the time of or close to the most severe pulmonary exacerbation experienced by the patient during the study period, were also studied. Sputum culture results (bacterial identification, load), clinical data [forced expiratory volume in 1 s (FEV_1_), body mass index, pulmonary exacerbations/stable status], and antibiotic courses were recorded.

### Antimicrobial Susceptibility Testing

Antimicrobial susceptibility testing was performed according to the 2016 recommendations of the Antibiogram Committee of the French Society for Microbiology^1^. Disk diffusion method was performed for amikacin, amoxicillin +/- clavulanic acid, aztreonam, cefalotin, cefepime, cefoxitin, cefpirom, cefotaxime, ceftazidime, chloramphenicol, ciprofloxacin, colistin, cotrimoxazole, fosfomycin, gentamicin, imipenem, isepamicin, levofloxacin, moxalactam, nalidixic acid, netilmicin, ofloxacin, piperacillin, piperacillin/tazobactam, tetracycline, ticarcillin +/- clavulanic acid, and tobramycin (Bio-Rad^®^). E-test (bioMérieux^®^) was performed when inhibition zone diameters were observed around disks, and minimal inhibitory concentration (MIC) results that differed by more than two dilutions were considered as different. For ciprofloxacin, MICs were determined by the agar dilution reference method.

### Motility and Biofilm Formation

Motility of *Pandoraea* isolates was tested in semi-solid agar according to Kirov et al. (2004). Biofilm formation was evaluated after 72 h of growth for *Pandoraea* isolates and after 48 h of growth for *P. aeruginosa* and *S. aureus* at 37°C in microtitre plates NUNC^®^-polystyrene (Abdouchakour et al., 2015). Isolates were categorized as non-adherent or weakly, moderately, or strongly adherent according to Stepanovic et al. (2000). Experiments were performed in triplicate.

### Virulence Assay in *Caenorhabditis elegans*

Virulence assays were performed in *Caenorhabditis elegans* model as previously described (Mosser et al., 2015), except that nematode growth medium (NGM) agar plates were inoculated with bacterial culture grown in Trypticase Soy Broth (TSB) for 24 h and incubated at 37°C for 28 h. Each experiment, including 2–3 plates per strain, was conducted once, except for *Escherichia coli* OP50, avirulent in *C. elegans* model, and *Pandoraea* Pp14′ that were included in each of the four experiments conducted during the study. The effect of *Pandoraea* culture supernatant on virulence and biofilm formation of two co-colonizing isolates (*S. aureus* Sa14 and *P. aeruginosa* Pa13) was tested by replacing TSB used for the initial culture of Sa14 and Pa13 by a mix of TSB 2X and *Pandoraea* culture supernatant (0.22 μm filtration of 48 h culture in TSB at 37°C) (vol/vol).

### Gene Sequencing and Genome Fingerprinting

16S rRNA and *gyrB* gene sequencing were performed as previously described (Carlier et al., 2002; Coenye and LiPuma, 2002). Pulsed-field gel electrophoresis (PFGE) after DNA macrorestriction by *Xba*I (Dupont et al., 2015), 16S rRNA gene PCR-temporal temperature gel electrophoresis (TTGE) (Dupont et al., 2015), and Enterobacterial repetitive intergenic consensus sequence-based PCR (ERIC-PCR) fingerprints (Atkinson et al., 2006) were performed as previously described. Pulsotype clustering analysis was based on Dice coefficient and Unweighted Pair Group Method with Arithmetic mean (UPGMA)^2^. Simpson’s Index of Diversity and Adjusted Wallace Coefficient (AWC) were calculated by using Comparing Partitions^3^. AWC was used to evaluate the degree of congruence among all isolates’ features.

### Statistical Analysis

Data were analyzed using GraphPad Prism version 5.00 for Windows (GraphPad Software, San Diego, CA, United States). Comparisons of nematode survival curves in presence of *Pandoraea* or co-colonizing *S. aureus* and *P. aeruginosa*, as well as virulence assays in presence of *Pandoraea* supernatant were performed using Log-rank tests. A *P*-value ≤ 0.05 was considered significant.

### Ethics Statement

Ethics approval was obtained through the Institutional Review Board of the University hospital of Nîmes (Interface Recherche Bioéthique IRB n°16.1101) for this observational study that fell within routine practice with non-additional diagnostic and monitoring procedures applied to the patient and retrospective analysis of primary data derived from routine clinical care.

## Results

### Genetic Relatedness and Variations among *Pandoraea* Isolates

Thirty-one *Pandoraea* isolates were recovered from 18 sputum samples (2–3 colonial morphotypes differing by their size, color and/or form and visually detected during the routine analysis of the sputum samples were analyzed for 10 samples) over a 7-year period of colonization (May 2008–February 2015) in a 22-year-old CF male patient. Partial 16S rDNA sequences of 1445 bp were identical for 29 isolates, the two remaining sequences (isolates Pp6 and Pp8) differing by one identical single nucleotide. The most related 16S rDNA sequences for type strains were those of *P. pulmonicola* CCUG 38759^T^ (>99.6% identity) and *Pandoraea pnomenusa* CCUG 38742^T^ (99.2%). Partial *gyr*B gene sequences (672 bp) were identical for 30 isolates, the sequence of isolate Pp14′ displaying one point mutation. The *gyr*B sequences presented 93.5% identity with that of the closest species *P. pulmonicola* strain DSM 16583^T^, a value compatible with the intraspecific variability described in other *Pandoraea* species (Coenye and LiPuma, 2002). These results suggested that the 31 isolates belonged to *P. pulmonicola*.

**FIGURE 1:**
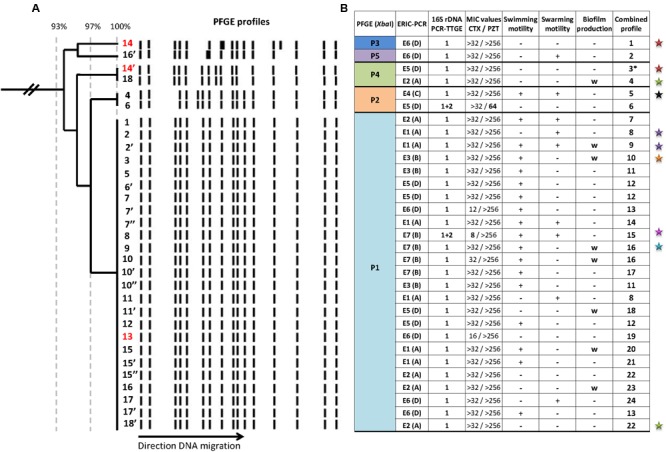
Genomic and phenotypic variability among 31 *Pandoraea pulmonicola* isolates recovered from 18 sputum specimens sampled over a 7-year period in a CF patient. **(A)** Genomic diversity of *Xba*I-generated pulsotypes obtained for sequential isolates assessed by clustering analysis based on Dice coefficient and Unweighted Pair Group Method with Arithmetic mean (UPGMA). The Dice’s coefficient scale is shown at the top of the dendrogram. Isolates were named with numbers (1–18) according to chronological sampling order, and isolates with different colonial morphotypes in a sample were named with the same number followed by apostrophe(s) (for example 10, 10′, 10″ correspond to three morphotypes recovered from the 10th sample out of 18). The supernatants of the three isolates noted in red (13, 14, and 14′) were included in the virulence assays in *Caenorhabditis elegans* model. **(B)** Summary of variability observed for genotypic and phenotypic characteristics. Isolates are in the same vertical order as in **(A)**. P1 to P5 are the different pulsotypes observed after *Xba*I-macrorestriction and pulsed-field gel electrophoresis (PFGE). E1 to E7 are the different profiles observed by enterobacterial repetitive intergenic consensus sequence-based PCR (ERIC-PCR). A to D corresponded to ERIC-PCR clusters formed by very related profiles differing by no more than two bands. Banding patterns obtained by 16S rDNA PCR-temporal temperature gel electrophoresis (TTGE) were named according to the bands observed as follows: 1 for profile composed by the unique band 1, and 1+2 for profile composed by the two bands 1 and 2. Minimal inhibitory concentrations (MICs) were given for cefotaxime (CTX) and piperacillin-tazobactam (PZT) in mg/L and bold type indicated values differing by more than two dilutions from others. The combined profile was established on the basis of the results presented in the seven preceding columns; if the seven traits were common, isolates were assigned the same combined profile. Stars indicated isolates recovered at the time of an episode of pulmonary exacerbation (one color per episode). +, positive; –, negative; w, weak. ^∗^, isolate with combined profile 3 also diverged by a point mutation in the *gyrB* sequence.

Molecular fingerprints revealed the *P. pulmonicola* isolates to be clonally related thereby confirming the chronic colonization (**Figure 1A**). Main pulsotype P1 was observed for 25 isolates while 4 other pulsotypes differing by no more than 5 DNA fragments from pulsotype P1, were each observed for one or two isolates (**Figure 1**). ERIC-PCR appeared more discriminative (seven profiles, E1 to E7, and Simpson’s Index of Diversity, SID = 0.862) than PFGE (SID = 0.351) but non-congruent with PFGE clusterisation (AWC = 0.003) (**Figure 1B**). The grouping of closely related ERIC-PCR profiles in clusters named A to D did not enhance the congruence with PFGE profiles clusterisation (**Figure 1B**). The 16S rRNA gene PCR-TTGE showed that a majority of isolates (29/31) harbored identical V3 region in all *rrs* copies. Copies with sequences differing by a guanine base addition (position 389 in the V3 region, *E. coli* K12 numbering; Sundquist et al., 2007) were found in the isolates Pp6 and Pp8 (**Figure 1B**), isolates which displayed in consequence different 16S rDNA sequences.

### Phenotype-Wide Variations among *P. pulmonicola* Isolates

All isolates were multidrug resistant. For most antibiotics, no inhibition zone diameters were observed around disks. MICs were determined for piperacillin-tazobactam, cefotaxime, imipenem, cotrimoxazole, and ciprofloxacin. Differences of two dilutions or more were observed for cefotaxime MIC for Pp8 and for piperacillin-tazobactam MIC for Pp6 compared with MIC values obtained for other isolates (**Figure 1B**). For the three other antibiotics, MIC values were either identical or did not differ by more than one dilution and were not considered as significant differences in antimicrobial susceptibility; particularly, MICs of ciprofloxacin were 32 mg/L (10 isolates) or 64 mg/L (21 isolates). Resistance traits (MICs) were congruent with 16S rRNA gene PCR-TTGE profiles (AWC = 1.00). Swimming and swarming motilities were observed for 58 and 29% of the isolates, respectively. Eight *P. pulmonicola* isolates (26%) were weak biofilm producers, the others being non-adherent. In most cases (except Pp14/14′ and Pp10′/10″), different colonial morphotypes in the same sample differed in their ability to swim, to swarm or to adhere (**Figure 1B**). Motility and adhesion were unrelated to other traits, such as resistance or genotype (AWC < 0.1).

### Overall Variability Trends

Variability was observed for all the characteristics studied, either for isolates recovered from serial samples but also between all isolates showing distinct colonial morphotypes within a sample. When results were combined, 24 sub-types were identified for the 31 clonally related isolates, highlighting that studied *Pandoraea* population was highly diverse (**Figure 1B**). The only association observed between studied characteristics was that the two isolates Pp6 and Pp8 (displaying 16S rDNA differing by one identical single nucleotide and intragenomic heterogeneity) were the only isolates with distinct antibiotypes. No chronological trend was observed for phenotypic and genotypic feature variability (**Figure 1**).

### Virulence and Co-virulence

**FIGURE 2:**
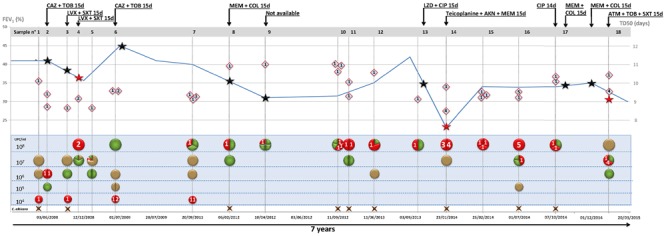
Diversity of cultivable pathogens, *Pandoraea* pulsotype, and virulence according to patient clinical status and antimicrobial courses. Data are presented horizontally for the 7-year period of patient clinical and microbiological follow-up and time scale indicated at the bottom of the figure (dd/mm/yyyy). For each of the 18 sputum samples, data are presented vertically. Bacteria are indicated according to load [left scale (top) in colony forming unit (CFU)/ml] and color code: *P. pulmonicola*, red circle; *P. aeruginosa*, green circle; *S. aureus*, gold circle. Brown cross at the bottom indicates the presence of *Candida albicans*. Segmented circles indicate the relative representation of different species (bicolor) or of different colonial morphotypes (unicolor). Lozenges represent life span of *C. elegans* fed with *Pandoraea* isolate (time to death 50, TD50, scale to the right). Numbers 1–5 in circles and diamonds indicate the pulsotype of corresponding *Pandoraea* isolate. The curve represents forced expiratory volume in 1 s [FEV_1_, left scale (bottom) (%)]. Stars indicate exacerbation periods and red stars exacerbation episodes with isolation of *Pandoraea* isolates with different pulsotypes. Black arrows indicate antibiotic courses (antimicrobial agents and treatment length in days are indicated on the arrow). AKN, amikacine; ATM, aztreonam; CAZ, ceftazidime; CIP, ciprofloxacine; COL, colistin; LVX, levofloxacine; LZD, linezolid; MEM, meropenem; SXT, cotrimoxazole; TOB, tobramycine.

For all *P. pulmonicola* isolates, the survival time of *C. elegans* was lower than that observed for the *E. coli* control strain with time required to kill 50% of the worm population or time to death 50 (TD50) in four experiments ranging from 11.2 to 12 days (mean time: 11.5 days, standard deviation: 0.4) (data not shown). Isolates exhibited variable virulence levels toward *C. elegans* since TD50 ranged from 8.4 to 11.1 days (average 9.8 days) (**Figure 2**). The relation between virulence expressed in nematode, isolation date, isolate sub-type and clinical status appeared unclear, except for the most virulent isolate Pp14′ (TD50 in four experiments ranging from 7.8 to 8.9 days, mean time: 8.4 days, standard deviation: 0.5) recovered in January 2014 at the time of the most severe pulmonary exacerbation (**Figure 2**).

**FIGURE 3:**
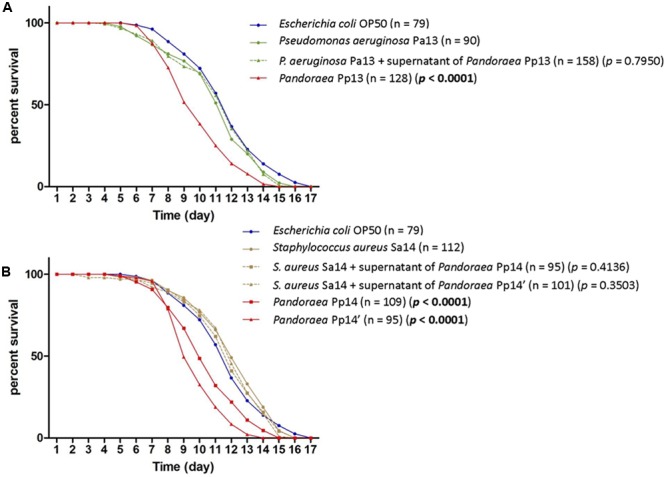
Survival curves of *C. elegans* fed either with *P. pulmonicola*, co-colonizing pathogen or co-colonizing pathogen grown in the presence of *Pandoraea* culture supernatant, compared to control (avirulent *Escherichia coli* OP50). **(A)** Worm life span for isolates recovered from sample 13 (September 2013): *Pseudomonas aeruginosa* Pa13, *P. pulmonicola* Pp13, and *P. aeruginosa* Pa13 cultured in the presence of *P. pulmonicola* Pp13 supernatant. n, number of nematodes tested per strain. *P*-value, statistical difference between the survival curve of the strain compared to that of *P. aeruginosa* Pa13 using Log-rank Test. **(B)** Worm life span for isolates recovered from sample 14 (January 2014, at the time of the most severe episode of pulmonary exacerbation experienced by the patient over the 7-year study period): *Staphylococcus aureus* Sa14, *P. pulmonicola* Pp14 and Pp14′, and *S. aureus* Sa14 grown in the presence of *P. pulmonicola* Pp14 or Pp 14′ supernatant. n, number of nematodes tested per strain. *P*-value, statistical difference between the survival curve of the strain compared to that of *S. aureus* Sa14 using Log-rank Test.

Both co-colonizing isolates of *S. aureus* (Sa14) and *P. aeruginosa* (Pa13) exhibited weak virulence in the nematode model (TD50 = 11.9 and 11.1 days, respectively), lower than those of *P. pulmonicola* Pp14, Pp14′, and Pp13, respectively grown from the same sample (*P*-value < 0.001) (**Figure 3**). Growth in the presence of *P. pulmonicola* supernatant did not affect the virulence of the co-colonizing isolates (*P*-value > 0.05) (**Figure 3**). Regarding biofilm formation, *P. aeruginosa* Pa13 was weak biofilm producer, while *S. aureus* Sa14 was moderately biofilm producer. No effects on *S. aureus* or *P. aeruginosa* biofilm production were observed in assays with *Pandoraea* supernatant (data not shown).

### Longitudinal Variability, Relation to Microbiological and Clinical Data

The patient is the only patient colonized by *Pandoraea* in our regional center caring for about 250 CF patients. He was diagnosed with CF at birth and is homozygous for the δ-F508 mutation. He was chronically colonized by *P. aeruginosa* and *S. aureus* and first acquired *Pandoraea* in May 2008. During the 7-year study period, the patient experienced 11 episodes of pulmonary exacerbation and received 12 courses of antibiotics (**Figure 2**). During the overall study period, a 10%-decrease of FEV_1_ was noted while body mass index increased from 14 to 20.5. The most severe pulmonary exacerbation occurred in January 2014 and was associated with significant pulmonary impairment (**Figure 2**).

*Pandoraea pulmonicola* was recovered from all the sputum sampled during the study period. The patient was also persistently colonized by *P. aeruginosa, S. aureus*, and *Candida albicans* but *P. pulmonicola* was the dominant bacterium or one of the dominant bacteria in 72% of the sputum samples. *P. pulmonicola* load ranged from 2.10^4^ to ≥10^8^ CFU/ml, being ≥10^8^ CFU/ml in 67% of the samples (**Figure 2**). Samples were analyzed at the time of 7 out of the 11 episodes of exacerbation. In five cases, *Pandoraea* was the dominant pathogen and bacterial load were ≥10^8^ CFU/ml in four cases. Isolates displaying minor pulsotypes were mostly recovered at the time of pulmonary exacerbation (4/6 isolates, 67%), including isolate Pp14′ which was non-motile, non-adherent, and more virulent in *C. elegans* than other *Pandoraea* isolates. Contrarily, swimming and swarming motilities were mainly observed for isolates not recovered during exacerbation (13/18 isolates, 72% and 5/9 stains, 55.5%, respectively). Weak biofilm formation, 16S rRNA gene sequence heterogeneity and distinct antibiotypes were equally observed for isolates recovered during exacerbation or stable clinical status and no relation between MIC values and antimicrobial courses received by the patient was noted. Particularly, no modification in MIC of ciprofloxacin was noted after the two documented antimicrobial courses including ciprofloxacin in September 2013 and October 2014. The five isolates that both swam and swarmed were isolated in the first year of patient colonization (**Figure 1B**) but adhesion, as well as other features, appeared unrelated to the date of isolation.

## Discussion

Bacterial diversification in CF niche has been described for the main gram-negative pathogens involved in the infectious pathogenesis of CF, *P. aeruginosa, Burkholderia* spp., and *Stenotrophomonas maltophilia* (Lieberman et al., 2014; Pompilio et al., 2016; Winstanley et al., 2016). We demonstrated herein that variability during persistence in the CFRT is also a main biological trait for the emerging pathogen *P. pulmonicola*. High intraclonal genotypic and phenotypic variability was highlighted with 24 different sub-types found for 31 isolates. All the variants observed herein in a single patient highlight a highly complex *P. pulmonicola* population probably involved in a patho-adaptive process. However, we did not observe a regular gradient of modifications between early and late isolates for a given trait or for combinations of traits as previously described during CF lung adaptation of other opportunistic pathogens (Hauser et al., 2011; Lieberman et al., 2014; Pompilio et al., 2016; Winstanley et al., 2016). Therefore, the detection of co-existing variants in the same specimen rather argues in favor of the presence in the CFRT of a population of variants. Coexisting variants are then detected or not depending on both their presence in the sample analyzed and their recognition during analysis. Such intra-population diversity, usually considered as conferring an adaptive ability to fluctuating environmental conditions, may thus be one of the major traits supporting *P. pulmonicola* adaptation to the CF lung (Lieberman et al., 2014; Winstanley et al., 2016).

All phenotypic characters studied herein are major patho-adaptive traits in the CF context (Hauser et al., 2011) and variability was observed for all of them. Regarding genotyping, serial *Pandoraea* isolates in a patient were previously shown to present related but variable profiles only when ERIC or BOX-PCR were used (Atkinson et al., 2006). Our study confirmed variability in ERIC-PCR profiles but is the first showing pulsotype diversity among clonally related persistent *Pandoraea* strains. Variations in PFGE profiles could be due to genome modifications such as mutations but, more probably, rearrangements, which were previously related to the patho-adaptation of *P. aeruginosa* in the CFRT (Kresse et al., 2003). As described previously for *Achromobacter* sp. strains persisting in CFRT, ERIC-PCR detected more variations than PFGE suggesting a genome flexibility involving small modules not detected by macrorestriction (Dupont et al., 2015). *rrn* intragenomic heterogeneity has also been described in adaptive evolution of other CFRT persisters, notably *Achromobacter xylosoxidans* and *S. maltophilia* (Michon et al., 2012). It generally leads to concerted evolution by homologous recombination between *rrn* that can promote chromosome rearrangements (Teyssier et al., 2003). Therefore, the heterogeneity of V3 *rrs* region and its dynamics assessed by PCR-TTGE has been considered herein as another witness of genomic events occurring in persistent *P. pulmonicola*. Finally, two comparative studies of whole genome sequences of successive *Pandoraea* CF clinical isolates are currently available showing that two *P. pnomenusa* isolates recovered 11 months apart did not reveal variability, except for one gene encoding a predicted phage tail protein (Ee et al., 2015) while three *Pandoraea apista* isolates recovered over a 1-year period accumulated mutations, deletions (up to 42 kb), and insertions (34.5 kb) over time (Greninger et al., 2017). These studies indicated that strain evolution within the CF lung may differ according the species and/or the host.

*Pandoraea pulmonicola* was reported as the predominant *Pandoraea* species among Irish CF patients (Costello et al., 2011) while *P. apista* is one of the most frequently isolated species from CF patients in United States (LiPuma, 2010). However, identification remains challenging in routine practice because mass spectrometry databases are incomplete and molecular identification may still be required for accurate knowledge on epidemiology and clinical implications of *Pandoraea* (Fernández-Olmos et al., 2012). In France, the first human case involving *P. pulmonicola* was reported in 2013 in a CF patient chronically colonized who died of *P. aeruginosa* sepsis (Kokcha et al., 2013). The epidemic spread of *P. pulmonicola* was also firstly described among six CF patients resulting in chronic colonization in the six patients who were also chronically colonized by *P. aeruginosa* (Degand et al., 2015); among them, three patients died, two patients remained clinically stable, and the remaining patient had a decline in lung function, as observed for the patient of the present study in whom dynamics of *P. pulmonicola* colonization may have been driven by antipseudomonal therapies (Kokcha et al., 2013). Regarding virulence, our results corroborated previous results suggesting that biofilm formation is not a major virulence factor in this genus (Caraher et al., 2008). A previous study showed that interactions occurred between *Pandoraea* spp. and *P. aeruginosa*, i.e., *P. pulmonicola* and *P. apista* growth was significantly inhibited by *P. aeruginosa* (Costello et al., 2014) and cellular components, as yet unidentified, rather than secreted factors were involved in such interactions (Costello et al., 2011). In the conditions of our study, evaluating effects of *Pandoraea* culture supernatant on *P. aeruginosa* and *S. aureus* virulence or biofilm formation, no interacting effects were observed. A previous study showed that virulence increased between two sequential *P. pulmonicola* isolates (Costello et al., 2011). Here, we found all the isolates to be mildly pathogenic in *C. elegans* model but at the time of the most severe exacerbation, one *Pandoraea* isolate was the most virulent isolate recovered during the study. If virulence factors remain largely unknown for *P. pulmonicola*, predicated virulence factors related to those in the genus *Burkholderia* were found in the *P. apista* type strain genome (Millard et al., 2015). Similarly, genes responsible for virulence, disease, and defense, were identified in *P. pnomenusa* genomes, with unique genes being identified in clinical isolates compared with environmental isolates (Ee et al., 2015).

*Pandoraea* are multidrug resistant bacteria; however, the genetic support for resistance and whether resistance is innate or acquired in this genus are still unknown. High-level resistance to antibiotics has previously been associated with fitness cost impacting various phenotypic traits including growth rate, virulence, and being involved in the emergence of bacterial diversity. Majority of available data come from studies on fluoroquinolones and the fitness costs associated with high-level fluoroquinolone resistance have been examined for a variety of pathogens: methicillin-resistant *S. aureus, Acinetobacter, E. coli, Salmonella, P. aeruginosa, Campylobacter, Clostridium*, …, and in the context of CF, for *Burkholderia cepacia* complex (Pope et al., 2008; Agnello et al., 2016; Fuzi, 2016). Studies showed complex and variable interplay between fluoroquinolone resistance and bacterial fitness depending on the species, the mutant, and the conditions, with either no measurable cost that may be related to the existence of compensatory mutations, reduction in biological fitness or enhanced fitness for the fluoroquinolone-resistant isolates (Marcusson et al., 2009; Redgrave et al., 2014). These data supported the determination of MIC of ciprofloxacine in this study but, in the absence of significant variability for this parameter, the impact of fluoroquinolone resistance and more largely of multidrug resistance on *Pandoraea* fitness and diversity has still to be explored.

Altogether, the results of our study prompt to sequence serial and unrelated *P. pulmonicola* CF isolates with the aim to get complementary information on nucleotide and gene content and dynamics, including genetic support for multidrug resistance, but also on whole genome structure and ultimately to better understand host–bacteria relationships.

## Note

This work was presented in part at the 29th Annual North American Cystic Fibrosis Conference, October 8-10, 2015, Phoenix, Arizona.

## Author Contributions

Conceived and designed the study: CD, FA, EJ-B, and HM; designed and performed the acquisition of clinical isolate collection: HM; performed the acquisition of clinical data: RC and PC; performed the acquisition of microbiological data: CD, FA, PC, and HM; performed the microbial analyses: CD and FA; analyzed and interpreted the data: CD, FA, EJ-B, and HM; drafted the paper: CD, FA, and HM; critically revised the manuscript: RC, PC, and EJ-B. All authors read and approved the final manuscript.

## Conflict of Interest Statement

The authors declare that the research was conducted in the absence of any commercial or financial relationships that could be construed as a potential conflict of interest.

## Footnotes

1 http://www.sfm-microbiologie.org/UserFiles/files/casfm/CASFM2016_V1_0_FEVRIER.pdf

2 http://genomes.urv.cat/UPGMA/

3 http://www.comparingpartitions.info/
